# Transducin-Like Enhancer of Split-1 Inhibits Malignant Behaviors *in vitro* and Predicts a Better Prognosis in Pancreatic Ductal Adenocarcinoma

**DOI:** 10.3389/fonc.2020.00576

**Published:** 2020-05-05

**Authors:** Yizhi Wang, Da Yuan, Li Zhou, Zhiyong Liang, Weixun Zhou, Jun Lu, Bolun Jiang, Lei You, Junchao Guo, Yu-Pei Zhao

**Affiliations:** ^1^Department of General Surgery, Peking Union Medical College Hospital, Chinese Academy of Medical Sciences, Peking Union Medical College, Beijing, China; ^2^Medical Management Office, Department of General Surgery, Peking Union Medical College Hospital, Chinese Academy of Medical Sciences, Peking Union Medical College, Beijing, China; ^3^Department of Pathology, Peking Union Medical College Hospital, Chinese Academy of Medical Sciences, Peking Union Medical College, Beijing, China

**Keywords:** pancreatic ductal adenocarcinoma, transducin-like enhancer of split-1, prognosis, survival, biomarker

## Abstract

**Background:** Transducin-like enhancer of split-1 (TLE1), a member of the Groucho/TLE family of transcriptional corepressors, has been reported to be involved in the tumorigenesis of various cancers and function as a clinical prognostic indicator. However, the mechanisms and prognostic significance of TLE1 in pancreatic ductal adenocarcinoma (PDAC) have not been elucidated.

**Methods:** In this study, western blot analyses and real-time polymerase chain reaction (RT-PCR) were employed to evaluate the expression of TLE1 and related proteins in PDAC cell lines. Wound healing, transwell migration and invasion, and Cell Counting Kit-8 (CCK-8) assays were used to determine cell line-specific differences in metastasis and proliferation. Flow cytometry was performed for cell cycle detection. RNA sequencing and bioinformatics were undertaken to explore the molecular mechanisms and potential targeted molecules of TLE1. TLE1 expression in tumor and para-tumor tissues was evaluated by tissue microarray-based immunohistochemistry using a semiquantitative method (*H*-score) in 262 patients with radical PDAC resection. Correlation, Kaplan–Meier survival, univariate, and multivariate analyses were also performed.

**Results:** Our findings showed that TLE1 expression was common in PDAC cell lines. Upregulation of TLE1 inhibited PDAC cell migration, invasion, and proliferation *in vitro* by delaying the G0/G1 transition. Immunohistochemistry revealed that TLE1 was specifically expressed in the nucleus and at higher levels in tumor tissues compared with para-tumor tissues. Generally, high TLE1 expression was associated with no vascular invasion. In univariate analyses, high TLE1 expression was associated with longer disease-specific survival (DSS) in all patients and in 16 patient subgroups. In multivariate analyses, TLE1 expression was independently associated with DSS in all patients and four patient subgroups.

**Conclusion:** In conclusion, these results suggest that TLE1 has an inhibitory role in PDAC progression and is a favorable prognostic indicator for patients with resectable PDAC.

## Introduction

Pancreatic ductal adenocarcinoma (PDAC) is becoming a leading cause of cancer-related death in both the United States and China because of its high mortality and extremely poor prognosis (1, 2). Given the poor prognosis of PDAC, understanding the factors that can influence and predict its prognosis is necessary. The conventional clinicopathological variables associated with PDAC prognosis include vascular invasion, perineural invasion, lymph node status, carbohydrate antigen 19-9 (CA19-9) level, and marginal status (3–7). Recently, several studies have explored associations between prognosis and the expression of genes that are involved in PDAC progression (8–10). Factors that are significantly associated with prognosis may have essential roles in PDAC diagnosis and targeted therapy. To date, several biomarkers that are associated with the prognosis of resectable PDAC have been identified (11–13). Additional promising and emerging biomarkers need to be further evaluated, and novel biomarker combinations may enhance prognostic efficiency.

Transducin-like enhancer of split-1 (TLE1), a corepressor belonging to the Groucho/TLE family, can modulate transcriptional output by binding to other transcription factors such as T cell factor/lymphoid-enhancing factor transcriptional factor (TCF/LEF) and RUNX to form multiprotein complexes. As such, TLE1 has important roles in development, including pancreatic development and neurogenesis (14–16). Recent studies have demonstrated a critical role for TLE1 in the progression of various tumors, such as synovial sarcoma, hematological malignancies, gastric cancer, and breast cancer (17–20). The nuclear factor kappa-B (NF-κB), Notch, and Wnt/β-catenin signaling pathways have been shown to be involved in TLE1-mediated modulation of tumors (21–23). Moreover, TLE1 can be a prognostic factor for various tumors. However, the roles of TLE1 in different tumors remain contradictory. Although TLE1 is generally regarded as an oncogene in several tumors, Di et al. described that TLE1 can serve as a tumor suppressor in hepatocellular carcinoma (24). However, to the best of our knowledge, no studies have explored clinical associations between TLE1 expression and PDAC. Therefore, this study aimed to clarify TLE1 expression in PDAC and investigate relationships between TLE1 expression and clinicopathological factors and prognosis in patients with resectable PDAC.

## Materials and Methods

### Cell Culture

Six human PDAC cell lines, AsPC-1, BxPC-3, PANC-1, Su86.86, T3M4, and MIA PaCa-2, were obtained from the American Type Culture Collection (Manassas, VA, USA) and grown in Dulbecco's modified Eagle's medium (DMEM) or Roswell Park Memorial Institute (RPMI) 1640 media (HyClone, Logan, UT, USA) supplemented with 10% fetal bovine serum (FBS) (Gibco, CA, USA) at 37°C in a 5% CO_2_ cell culture incubator.

### Transfection

Cells were seeded in 12-well plates and cultured for 24 h. When the cells reached 50% confluence, they were transfected with a TLE1 overexpression plasmid (termed OE), negative control plasmid (termed NOE), TLE1 knockdown plasmid (termed SH), or scrambled control plasmid (termed NSH) according to the manufacturer's instructions. The plasmids for TLE1 overexpression and knockdown were purchased from GenePharma (Shanghai, China) and YouBio (Changsha, China), respectively. The specificity and efficiency of transfections were validated by western blot and real-time polymerase chain reaction (RT-PCR) analyses in the transfected cell lines.

### Western Blot Analyses

Cells were lysed with radioimmunoprecipitation assay (RIPA) buffer (2500628; Merck Millipore Ltd., Billerica, MA, USA) supplemented with a proteinase inhibitor cocktail (P1265; Applygen Technologies Inc., Beijing, China) on ice for 30 min and centrifuged at 12,000 rpm for 10 min. The supernatants were collected and measured for their protein concentrations using a bicinchoninic acid (BCA) Protein Assay Kit (Thermo Fisher Scientific, Rockford, IL, USA). Aliquots of the lysates containing 20 μg of protein were loaded onto 10% Bis-Tris sodium dodecyl sulfate–polyacrylamide gel electrophoresis (SDS-PAGE) gels and electrophoresed at 80 V for 2.5 h. The separated proteins were transferred onto Immobilon-PVDF membranes (IPVH00010; Merck Millipore Ltd.) by electroblotting at 200 mA for 2.5 h in a transblot cell on ice. Membranes were blocked with 5% non-fat milk at room temperature for 1 h, incubated with primary antibodies anti-TLE1 antibody [1:1,000 dilution; EPR9386 (2); Abcam, Cambridge, UK], anti-β-actin antibody (1:1,000 dilution; C1313; Applygen Technologies Inc.,), anti-N-cadherin antibody (1:1,000 dilution; 13116; Cell Signaling Technology; Boston, MA, USA), anti-E-cadherin antibody (1:1,000 dilution; 14472; Cell Signaling Technology, Boston, MA, USA), anti-vimentin antibody (1:1,000 dilution; 5471; Cell Signaling Technology, Boston, MA, USA), anti-cyclin A2 antibody (1:1,000 dilution; ab137769, Abcam, Cambridge, UK), anti-cyclin B1 antibody (1:50,000 dilution; ab32053, Abcam, Cambridge, UK), and anti-cyclin D1 antibody (1:200 dilution; ab16663, Abcam, Cambridge, UK) overnight at 4°C; washed five times for 10 min each in TBS with 0.1% Tween (TBS-T) at room temperature; and incubated with anti-rabbit or anti-mouse secondary antibodies conjugated with peroxidase (1:1,000 dilution; C1309 [Rabbit] and C1308 [Mouse]; Applygen Technologies Inc.) in 5% non-fat milk for 2 h at room temperature. Finally, the membranes were washed five times for 10 min each in TBS-T at room temperature and developed with an enhanced chemiluminescence (ECL) detection system (Tanon 5500; Tanon, Shanghai, China).

### RNA Isolation and Real-Time Polymerase Chain Reaction

Cells were plated in 6-well plates at 5 × 10^5^ cells/well and transfected for 48 h. Total RNA was extracted from the transfected PDAC cells with TRIzol^®^ Reagent (15596026; Ambion, Life Technologies, Carlsbad, CA, USA) and subjected to first-strand cDNA synthesis with a First-Strand Synthesis System for RT-PCR (Takara, Tokyo, Japan). The obtained cDNAs were quantified by RT-PCR using a Veriti^®^ 96-Well Thermal Cycler (4375786; Applied Biosystems, Foster City, CA, USA). PCR was conducted using a StepOnePlus™ system (Applied Biosystems) in accordance with the manufacturer's instructions. The TLE1 primers were as follows: forward, 5′-CCTCCTACACAGCAGCAGTT-3′; reverse, 5′-TCTGCATCGTGGTGCTTCTT-3′. GAPDH was used as the reference gene, and the primers were as follows: forward, 5′-CGGAGTCAACGGATTTGGTCGTAT-3′; reverse, 5′-AGCCTTCTCCATGGTGGTGAAGAC-3′. Fold changes relative to GAPDH were calculated using −2^ΔΔCt^ method.

### Wound Healing and Transwell Migration and Invasion Assays

Cells were cultured in 6-well plates at 1 × 10^6^ cells/well in a medium without FBS. When the cells reached 70–80% confluence, they were wounded by scratching with a sterile pipette tip, washed at least three times with phosphate-buffered saline (PBS), and cultured in Opti-MEM^®^ (Gibco, Life Technologies, Beijing, China). At 0, 12, 24, 36, and 48 h after being wounded, the cells were observed and images were obtained using a DFC300FX microscope (Leica, Jena, Germany).

For transwell migration and invasion assays, we used non-coated or coated membranes in transwell chambers (24-well insert; 8-μm pore size; Corning Life Sciences, Corning, NY, USA). Cells (1 × 10^4^) were plated in the top chamber and cultured with no serum medium after transfection in 6-well plates for 24 h. A medium containing 10% FBS was added to the lower chamber. After incubation at 37°C in 5% CO_2_ for 36 h, unpenetrated cells in the upper chamber were wiped with a cotton swab, and the penetrated cells were fixed in methyl alcohol for 20 min and subjected to hematoxylin–eosin (H&E) staining 10 and 5 min, respectively, for counting. Cell numbers were counted under a DFC300FX microscope.

### Cell Counting Kit-8 Cell Proliferation Assay

For proliferation assays, cells were cultured in 96-well plates at 3 × 10^3^ cells/well in a medium supplemented with 10% FBS at 37°C in 5% CO_2_ after transfection in 6-well plates for 24 h. For the assays, 10 μl/well Cell Counting Kit-8 (CCK-8) reagent (Dojindo, Kumamoto, Japan) was added at 0, 24, 48, 72, and 96 h. After 2-h incubation at 37°C, the optical densities were measured at 450 nm (OD450) using a microplate reader (Wellscan MK3; Thermo Labsystems, Helsinki, Finland). OD630 values were measured as a reference.

### Cell Cycle Detection

When the cells reached 75–85% confluence, they were collected and fixed in 70% ethanol at −20°C for >18 h. The cells were then washed with PBS, centrifuged at 1,000 rpm for 5 min, and incubated with RNase A (0.2 mg/ml) in PBS. After propidium iodide was added to the cell suspension, the samples were evaluated by flow cytometry (BD Accuri™ C6 Plus; BD, San Jose, CA, USA).

### RNA Sequencing and Bioinformatics

RNA libraries were constructed using VAHTS Stranded mRNA-seq Library Prep Kit (Vazyme, Nanjing, JS, China), according to the manufacturer's instructions. Libraries were controlled for quality and quantified using the Bioanalyzer 2100 system (Agilent Technologies, Santa Clara, CA, USA). Libraries were reverse-transcribed into single-strand DNA molecules, captured on Illumina flow cells, amplified *in situ* as clusters, and finally sequenced for 150 cycles on an Illumina HiSeq Sequencer, according to the manufacturer's instructions. Raw data were generated after sequencing, image analysis, base calling, and quality filtering on an Illumina HiSeq 2500 sequencer. After adaptor trimming and removal of low-quality reads using cut adapt (v 1.9.2) software, high-quality reads were generated. These reads were aligned to the reference genome (UCSC hg19) guided by the Ensembl GFF gene annotation file (v 70) with hisat2 software (v 2.04). The cuffdiff software (part of cufflinks, v 2.2.1) was used to determine the levels of FPKM to compare mRNA expression profiles, and fold changes and *P*-values were calculated using FPKM to identify differentially expressed mRNA. Gene Ontology (GO) and Kyoto Encyclopedia of Genes and Genomes (KEGG) pathway enrichment analyses were performed based on the differentially expressed mRNAs.

### Patients and Tissue Samples

Tumor and para-tumor tissues were obtained from 262 patients who underwent radical surgical resection (R0) at Peking Union Medical College Hospital from December 2004 to September 2014. The diagnosis of PDAC was confirmed pathologically according to the World Health Organization criteria. This study was approved by the Institutional Ethics Committee. All patients signed written informed consent. The clinicopathological data collected from patients included demographic characteristics, tumor status, and complete follow-up data, all of which are summarized in Table 1.

**Table 1:** Relationships between TLE1 expression and clinicopathological characteristics in PDAC patients.

**Variables**	***n***		**TLE1 expression**	
		**High**	**Low**	*P*-value^*^
**Sex**				0.790
Female	124	73	51	
Male	138	79	59	
**Age**				0.456
≤ 60	119	72	47	
>60	143	80	63	
**Diabetes**				0.787
Absent	228	133	95	
Present	34	19	15	
**CA19-9 level**				0.222
Normal	43	29	14	
Elevated	173	99	74	
**Tumor size**				0.476
≤ 4 cm	193	108	85	
>4 cm	47	29	18	
**Tumor location**				0.188
Non-head	96	61	35	
Head	156	86	70	
**Histological grade**				0.243
G1–2	148	94	54	
G3–4	74	41	33	
**Perineural invasion**				0.606
Absent	74	46	28	
Present	51	34	17	
**Vessel invasion**				**0.022**
Absent	98	66	32	
Present	18	7	11	
**T stage**				0.159
T1–2	13	10	3	
T3	245	140	105	
**N stage**				0.701
N0	111	62	49	
N1	139	81	58	

* *χ^2^-test. The total patient number does not equal to 262 for all variables owing to a lack of patient information in some cases*.

*PDAC, pancreatic ductal adenocarcinoma; G1, well-differentiated; G2, moderately differentiated; G3, poorly differentiated; G4, undifferentiated; N, lymph node; T, tumor; TLE1, transducin-like enhancer of split-1. Bold values indicate the values are <0.05 which are statistically significant*.

### Tissue Microarray Construction

The tissue microarray (TMA) was constructed using formalin-fixed paraffin-embedded blocks of PDAC tissues after diagnosis by routine H&E staining-based pathological examination. Following review and selection of representative tumor regions, two cores for each patient were sampled using a 1.5-mm punch. TMA construction was performed with a manual tissue arrayer (Beecher Instruments, Sun Prairie, WI, USA).

### Immunohistochemistry

TLE1 expression was detected by immunohistochemistry using an anti-TLE1 monoclonal primary antibody (ab183742, Abcam, Cambridge, UK) and a two-step staining kit (EnVision™ Detection System; Dako, Copenhagen, Denmark). Tissues were cut cross-sectionally into 4-μm-thick sections, mounted, deparaffinized, and rehydrated. After antigen retrieval by autoclaving in 0.01 M of citrate buffer at 95°C for 10 min, the sections were incubated with 3% hydrogen peroxide to block endogenous peroxidase activity, followed by incubation with the primary antibody (1:100 dilution) overnight at 4°C. After being washed with PBS, the sections were incubated with a horseradish peroxidase-labeled secondary antibody (EnVision™ Detection System). Following further washing with PBS, sections were stained with diaminobenzidine and counterstained with hematoxylin. Normal non-immune rabbit serum (ab7487, Abcam) at the same dilution was applied as a negative control.

### Staining Evaluation

Two experienced pathologists (ZYL and WXZ) without prior knowledge of clinicopathological or survival data independently evaluated the staining. Discrepancies were resolved by joint evaluation to reach a consensus. The *H*-score, a widely used semiquantitative immunohistochemical evaluation method that assesses both the intensity and percentage of positively stained cells, was used for further evaluation. The range of *H*-scores is 0–300. A cutoff value for the *H*-score reflecting high/low TLE1 expression in tumors was determined by the largest Youden index within the receiver operating characteristic (ROC) curve, as described previously (25).

### Follow-Up and Evaluated Variables

Complete follow-up data were obtained for 229 patients (87.4%), with follow-up periods ranging from 1 to 62 months (median, 17 months). By the end of the follow-up, 84 patients were alive and 145 had died. Eleven clinicopathological variables were included for evaluation: sex, age, diabetes, CA19-9 level, tumor size, tumor location, histological grade, perineural invasion, vessel invasion, T stage, and N stage.

### Survival and Expression Analyses Using the Gene Expression Profiling Interactive Analysis and Kaplan–Meier Plotter Online Databases

Two online databases, Gene Expression Profiling Interactive Analysis (GEPIA) (http://gepia2.cancer-pku.cn) and Kaplan–Meier Plotter (www.proteinatlas.org), which are based on The Cancer Genome Atlas (TCGA) and Genotype-Tissue Expression (GTEx) program, were used to analyze survival according to protein and mRNA expression levels via the log-rank test. The GEPIA database was also used to evaluate differential expression between tumor tissues and para-tumor tissues.

### Statistical Analysis

*H*-scores for TLE1 staining in tumor and para-tumor tissues were compared by the Mann–Whitney *U*-test. The χ^2^-test was used to evaluate associations between TLE1 expression and clinicopathological variables. The prognostic significance of TLE1 expression was evaluated by the Kaplan–Meier method and the log-rank test. Cox regression (proportional hazards model) was employed for univariate and multivariate analyses of prognostic factors. Differences between groups were analyzed by Student's *t*-test. All statistical analyses were performed using SPSS 22.0 software (IBM Inc., Armonk, NY, USA). Values of *P* < 0.05 were considered to indicate statistically significant differences.

## Results

### TLE1 Expression in Pancreatic Ductal Adenocarcinoma Cell Lines

**Figure 1:**
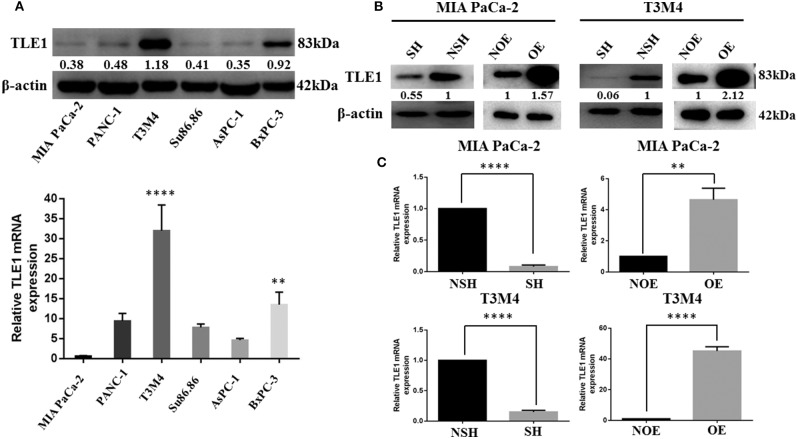
Selection of MIA PaCa-2 and T3M4 cell lines as the experimental models and establishment of cell lines by transient transfection for TLE1 overexpression or TLE1 knockdown. **(A)** TLE1 protein and mRNA expression in six PDAC cell lines. **(B,C)** The efficacies of TLE1 overexpression and TLE1 knockdown were confirmed by western blot and RT-PCR analyses. Data are presented as mean ± SD. ^**^P < 0.01, ^****^P < 0.0001, by Student's *t*-test. PDAC, pancreatic ductal adenocarcinoma; TLE1, transducin-like enhancer of split-1; OE, TLE1 overexpression; NOE, negative control for TLE1 overexpression; SH, TLE1 knockdown; NSH, scrambled control (*n* = 3).

Six PDAC cell lines were used to explore TLE1 expression, which was observed in all six cell lines by western blot analyses, but significant differences existed among these cell lines (Figure 1A). Specifically, T3M4 cells had the highest TLE1 expression followed by BxPC-3 cells, whereas the differences among the other PDAC cell lines were non-significant. MIA PaCa-2 cells had the lowest TLE1 expression. These expression differences were further confirmed by RT-PCR (Figure 1A). Based on these results, T3M4 and MIA PaCa-2 cells were selected for further analysis. Subsequently, TLE1 overexpression (TLE1-OE) and knockdown (TLE1-SH) cell lines were established by transient transfection of the T3M4 and MIA PaCa-2 cell lines. Transfection efficacies in the TLE1-OE and TLE1-SH cell lines were compared with the corresponding control cell lines and confirmed by western blot and RT-PCR analyses (Figures 1B,C).

### TLE1 Inhibits Pancreatic Ductal Adenocarcinoma Cell Metastasis Ability *in vitro*

**Figure 2:**
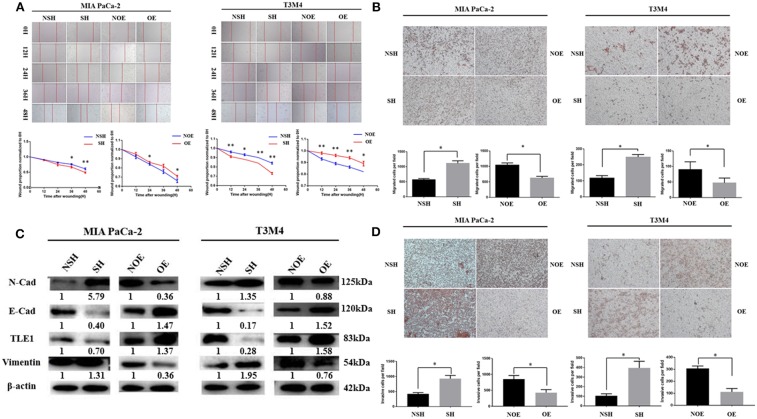
TLE1 inhibits PDAC cell migration and invasion *in vitro*. **(A)** PDAC cell migration capabilities were evaluated by wound healing assays in MIA PaCa-2 and T3M4 cells (magnification: 100×). **(B)** PDAC cell migration abilities were assessed by transwell assays in MIA PaCa-2 and T3M4 cells (magnification: 100×). **(C)** PDAC cell invasion abilities were assessed by transwell assays in MIA PaCa-2 and T3M4 cells (magnification: 100×). **(D)** TLE1 overexpression increased E-cadherin expression and decreased N-cadherin and vimentin expression, whereas TLE1 knockdown had the opposite effects. Data are presented as mean ± SD. ^*^P < 0.05, ^**^P < 0.01, by Student's *t*-test. TLE1, transducin-like enhancer of split-1; PDAC, pancreatic ductal adenocarcinoma; OE, TLE1 overexpression; NOE, negative control for TLE1 overexpression; SH, TLE1 knockdown; NSH, scrambled control (*n* = 3).

*In vitro* wound healing and transwell migration and invasion assays using the TLE1-OE and TLE1-SH cell lines were employed to evaluate the effects of TLE1 on cell metastasis ability. Wound healing assays revealed that, compared with control cells, TLE1 overexpression significantly prolonged wound healing time, whereas TLE1 knockdown significantly shortened wound healing time in both T3M4 and MIA PaCa-2 cell lines (Figure 2A). Then we used transwell migration and invasion assays to further confirm these findings. TLE1 overexpression significantly impaired the migration and invasion capacity of T3M4 and MIA PaCa-2 cells, whereas TLE1 knockdown significantly accelerated cell migration and invasion (Figures 2B,C). On the basis of the role of TLE1 in tumor metastasis ability, we further explored the effects of TLE1 on epithelial–mesenchymal transition (EMT), which plays an essential role in tumor cell migration and invasion. TLE1 knockdown significantly increased the expression of the mesenchymal markers N-cadherin and vimentin, whereas TLE1 overexpression increased expression of the epithelial marker E-cadherin (Figure 2D).

### TLE1 Hampers Pancreatic Ductal Adenocarcinoma Cell Proliferation by Enhancing G0/G1 Retention

**Figure 3:**
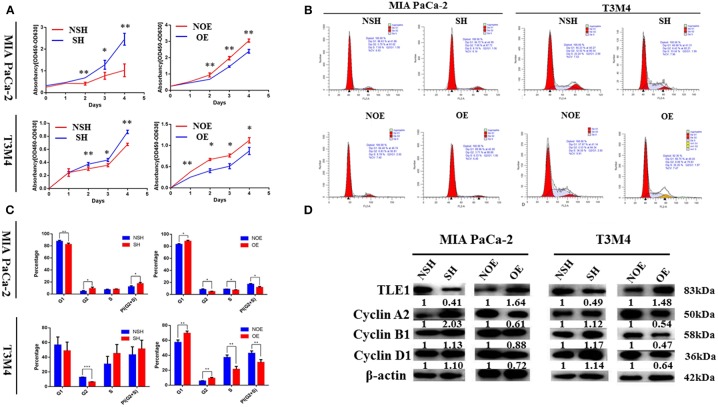
TLE1 hampers PDAC cell proliferation and cell cycle progression *in vitro*. **(A)** PDAC cell proliferation was evaluated by CCK-8 assays in MIA PaCa-2 and T3M4 cells. **(B,C)** Cell cycle progression was detected using flow cytometry, and the percentages of G1, G2, and S phase cells in the MIA PaCa-2 and T3M4 cell lines were calculated. **(D)** Levels of critical cell cycle regulators were evaluated by western blot. Data are presented as mean ± SD. ^*^P < 0.05, ^**^P < 0.01, ^***^P < 0.001, by Student's *t*-test. CCK-8, Cell Counting Kit-8; TLE1, transducin-like enhancer of split-1; PDAC, pancreatic ductal adenocarcinoma; OE, TLE1 overexpression; NOE, negative control for TLE1 overexpression; SH, TLE1 knockdown; NSH, scrambled control (*n* = 3).

CCK-8 assays were used to analyze the effects of TLE1 on cell proliferation. From day 2, significant differences in cell proliferation rates appeared between experimental and control cells in both cell lines. TLE1 overexpression significantly reduced the proliferation capacity of both cell lines, whereas TLE1 knockdown significantly enhanced proliferation (Figure 3A). To thoroughly investigate the underlying mechanisms, we used flow cytometry to evaluate the influence of TLE1 on cell cycle progression. TLE1 overexpression increased the ratio of G0/G1 phase cells and decreased the ratio of G2/M and S phase cells, leading to a lower proliferation index, whereas TLE1 knockdown produced the opposite findings (Figures 3B,C). These results suggested that TLE1 can impede the G1/S transition. Western blot analyses were performed to evaluate the expression of cell cycle-related proteins. Upon TLE1 overexpression, both the G1/S marker cyclin D1 and the G2/M markers cyclin B1 and cyclin A2 were decreased, whereas levels of all three proteins were increased by TLE1 knockdown (Figure 3D).

### Bioinformatics for Potential Targets of TLE1

**Figure 4:**
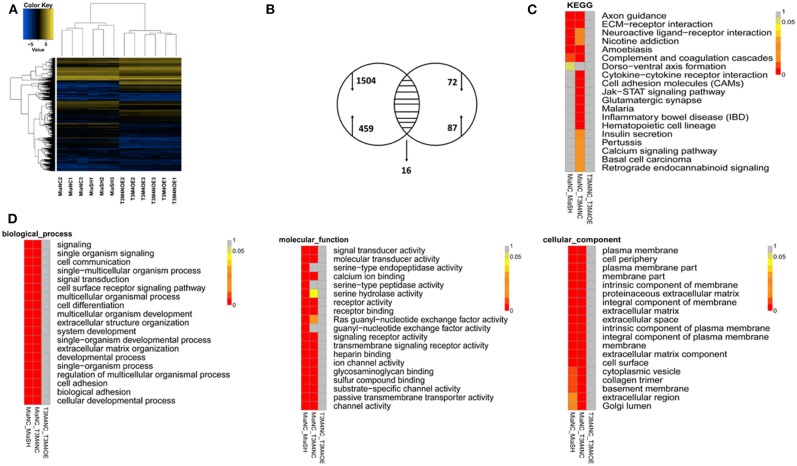
Bioinformatic screening for potential targeted molecules of TLE1. **(A)** Gene transcription profiles of genes that were positively or negatively regulated by TLE1 in MIA PaCa-2 and T3M4 cells. **(B)** Aberrant expression of TLE1-targeted genes in MIA PaCa-2 and T3M4 cells included in a Venn diagram. **(C)** The Kyoto Encyclopedia of Genes and Genomes (KEGG) pathway analyses for TLE1-regulated genes. **(D)** The representative Gene Ontology (GO) pathway analysis result for TLE1-regulated genes.

To further explore the molecular mechanisms of TLE1 regulating the malignant biological behavior of PDAC, TLE1 expression was knockdown in MIA PaCa-2 cells, and TLE1 expression was upregulated in T3M4 cells. The RNA transcriptome was used to establish a transcription profile of TLE1 downstream target genes. The results showed that 16 mRNAs were regulated by TLE1 in both the silenced and overexpression groups, including *SNCB, SLC17A7, LYL1, EGR4, DISC1, LINC00982, LINC00304, AP003774.1, MMP23B, IGLC2, IGHA1, LINC01786, C4orf48, AP000344.2, AL845552.2*, and *SRXN1* (Figures 4A,B). GO analysis and KEGG analysis revealed that these 16 genes are involved in cell biological processes, cell composition, and molecular functions. Specifically, these genes regulated by TLE1 are involved in signaling pathways such as naive CD8+ T cells, calcineurin-regulated NFAT-dependent transcription in lymphocytes, and EMT, which indicated that TLE1 may participate in biological processes such as immune regulation and tumor metastasis (Figures 4C,D).

### Characteristics of TLE1 Expression in Pancreatic Ductal Adenocarcinoma Samples

**Figure 5:**
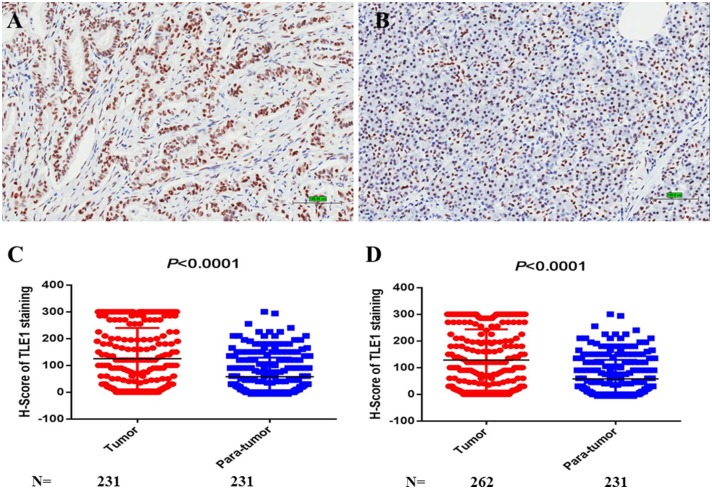
TLE1 expression in pancreatic ductal adenocarcinoma (PDAC). **(A)** TLE1 expression in tumor tissues (original magnification: 200×; bar, 100 μm). **(B)** TLE1 expression in para-tumor tissues (original magnification: 200×; bar, 100 μm). **(C)** Comparison of *H*-scores for TLE1 expression between tumor and paired para-tumor tissues (*P* < 0.0001; Mann–Whiney *U*-test). **(D)** Comparison of *H*-scores for TLE1 expression between tumor and non-paired para-tumor tissues (*P* < 0.0001; Mann–Whiney *U*-test) (*n* = 3).

Positive TLE1 staining was observed in the nuclei of both tumor and para-tumor tissues (Figures 5A,B). The median *H*-scores were 100 (range, 0–300) in tumor tissues and 25 (range, 0–300) in para-tumor tissues. With the use of the Mann–Whitney *U*-test, the *H*-score of tumor tissues was significantly higher than that of para-tumor tissues in both the paired and non-paired tumor and para-tumor tissues. These data generally coincided with the results from the GEPIA database but without a significant difference in GEPIA database (*P* < 0.0001; Figures 5C,D and Figure S1A).

### Cutoff Value for the TLE1 *H*-Score

**Figure 6:**
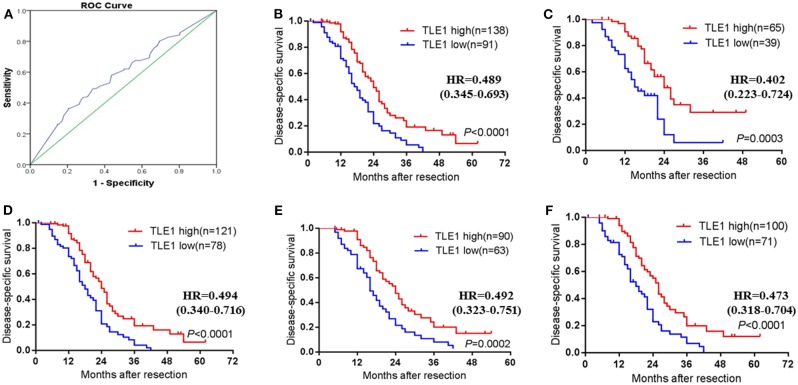
Prognostic impact of TLE1 expression in the whole PDAC patient cohort and selected patient subgroups. **(A)** ROC curve for tumor *H*-scores of TLE1 staining for DSS status and the cutoff value. **(B)** DSS curve for all patients with high or low tumor TLE1 expression (*P* < 0.0001; log-rank test). **(C)** Patients aged ≤ 60 years (*P* = 0.0003; log-rank test). **(D)** Non-diabetic patients (*P* < 0.0001; log-rank test). **(E)** Patients with elevated CA19-9 (*P* = 0.0002; log-rank test). **(F)** Patients with tumor size ≤ 4 cm (*P* < 0.0001; log-rank test). DSS, disease-specific survival; CA19-9, carbohydrate antigen 19-9; TLE1, transducin-like enhancer of split-1; PDAC, pancreatic ductal adenocarcinoma; ROC, receiver operating characteristic (*n* = 3).

After the ROC curve was analyzed (Figure 6A), the cutoff value for the TLE1 *H*-score was identified as the largest Youden index for survival status (72.5).

### Associations of TLE1 Expression With Clinicopathological Variables in Pancreatic Ductal Adenocarcinoma

Among the 11 clinicopathological variables evaluated, high TLE1 expression was significantly associated with absent vascular invasion (*P* = 0.022; Table 1). TLE1 expression was not significantly associated with the other 10 clinicopathological variables (*P* > 0.05).

### Prognostic Significance of TLE1 in Pancreatic Ductal Adenocarcinoma After Radical Resection

The Kaplan–Meier survival curve showed that high TLE1 expression was significantly associated with improved disease-specific survival (DSS), in accordance with the results from the Kaplan–Meier Plotter online database (*P* < 0.001; Figure 6B and Figure S1B). In univariate analyses, TLE1 expression was a prognostic indicator for DSS (*P* < 0.001; Table 2). *N* stage and vascular invasion were also prognostic indicators in univariate analyses (*P* < 0.05; Table 2). However, in the multivariate analysis, only TLE1 expression was confirmed as an independent prognostic factor (*P* = 0.001; Table 2).

**Table 2:** Univariate and multivariate analyses for prognostic factors in resectable PDAC.

**Variables**	**Number (*n*)**		**Univariate analysis**		**Multivariate analysis**		
		**HR**	**95% CI**	*P* value^*^	**HR**	**95% CI**	***P* value^#^**
							
**Sex**				0.559			
Female	109	1					
Male	120	1.104	0.793–1.535				
**Age**				0.924			
≤ 60	105	1					
>60	124	0.984	0.703–1.376				
**Diabetes**				0.934			
Absent	199	1					
Present	30	0.978	0.580–1.649				
**CA19-9 level**				0.377			
Normal	39	1					
Elevated	153	1.228	0.778–1.938				
**Tumor size**				0.322			
≤ 4 cm	171	1					
>4 cm	38	1.247	0.806–1.930				
**Tumor location**				0.961			
Non-head	89	1					
Head	131	0.992	0.703–1.398				
**Histological grade**				0.066			
G1–2	127	1					
G3–4	67	1.424	0.977–2.075				
**Perineural invasion**				0.135			
Absent	67	1					
Present	46	1.495	0.883–2.531				
**Vessel invasion**				**0.010**			0.055
Absent	88	1			1		
Present	16	2.542	1.245–5.193		2.030	0.984–4.186	
**T stage**				0.233			
T1–2	13	1					
T3	213	1.590	0.742–3.406				
**N stage**				**0.008**			0.199
N0	94	1			1		
N1	126	1.606	1.129–2.285		1.417	0.833–2.410	
**TLE1 expression**				**<0.001**			**0.001**
Low	91	1			1		
High	138	0.545	0.391–0.759		0.398	0.236–0.671	

*The total patient number does not equal to 262 for all variables owing to a lack of patient information in some cases*.

*G1, well-differentiated; G2, moderately differentiated; G3, poorly differentiated; G4, undifferentiated; HR, hazard ratio; N, lymph node; PDAC, pancreatic ductal adenocarcinoma; T, tumor; TLE1, transducin-like enhancer of split-1; 95% CI, 95% confidence interval*.

* *Log-rank test*.

# *Multivariate Cox regression. Bold values indicate the values are <0.05 which are statistically significant*.

In subgroup survival analyses, more than half of the subgroups (namely, female, male, age ≤ 60 years, age > 60 years, non-diabetic, elevated CA19-9, small tumor size (≤ 4 cm), head and non-head tumor locations, G3–G4 tumor grade, absence and presence of perineural invasion, absent vascular invasion, T3 stage, N0 stage, and N1 stage, and TLE1 expression) were prognostic factors in univariate analyses (*P* < 0.05; Table 3). However, in the multivariate analysis, TLE1 expression remained significantly different in only four subgroups: age ≤ 60 years, non-diabetic, elevated CA19-9, and small tumor size (≤ 4 cm) (*P* < 0.05; Table 3). Survival curves are shown in Figures 6C–F. TLE1 expression was also a marginal prognostic indicator in the subgroups female, age > 60 years, non-head tumor location, and G3–G4 tumor grade in the multivariate analysis (0.05 < *P* < 0.1; Table 3).

**Table 3:** Prognostic value of TLE1 expression in selected PDAC patient subgroups.

**Variables**	**Univariate analysis**			**Multivariate analysis**		
	**HR**	**95% CI**	***P*-value**	**HR**	**95% CI**	***P*-value**
Female	0.527	0.329–0.845	**0.008**	0.379	0.141–1.019	0.054
Male	0.460	0.289–0.731	**0.001**	0.417	0.120–1.444	0.167
Age ≤ 60	0.382	0.226–0.646	**<0.001**	0.188	0.045–0.791	**0.023**
Age > 60	0.558	0.364–0.855	**0.007**	0.550	0.286–1.057	0.073
Non-diabetic patients	0.486	0.342–0.690	**<0.001**	0.547	0.368–0.814	**0.003**
Elevated CA19-9	0.477	0.319–0.713	**<0.001**	0.561	0.355–0.887	**0.013**
Tumor ≤ 4 cm	0.465	0.318–0.681	**<0.001**	0.446	0.257–0.774	**0.004**
Non-head location	0.437	0.254–0.753	**0.003**	0.510	0.258–1.009	0.053
Head location	0.527	0.341–0.813	**0.004**	0.469	0.15–1.623	0.232
G3–4 grade	0.413	0.227–0.751	**0.004**	0.342	0.095–1.223	0.099
Absent perineural invasion	0.458	0.240–0.875	**0.018**	0.663	0.268–1.642	0.375
Present perineural invasion	0.418	0.183–0.954	**0.038**	0.566	0.116–2.765	0.482
Absent vessel invasion	0.431	0.244–0.762	**0.004**	0.621	0.263–1.467	0.278
T3 stage	0.500	0.356–0.703	**<0.001**	0.719	0.321–1.612	0.424
N0 stage	0.385	0.228–0.652	**<0.001**	0.663	0.207–2.118	0.487
N1 stage	0.571	0.363–0.898	**0.015**	0.490	0.152–1.576	0.231

*G3, poorly differentiated; G4, undifferentiated; N, lymph node; HR, hazard ratio; PDAC, pancreatic ductal adenocarcinoma; T, tumor; TLE1, transducin-like enhancer of split-1; 95% CI, 95% confidence interval. Bold values indicate the values are <0.05 which are statistically significant*.

## Discussion

As a member of the Groucho/TLE family, TLE1 consists of five conserved domains, Q, GP, CcN, SP, and WD40, among which, Q and WD40 are the main functional domains (14). TLE1 is involved in many signaling pathways that regulate different cellular functions and is modulated by various mechanisms. In Wnt/β-catenin signaling pathway, targeted gene transcription is activated by β-catenin interactions with sequence-specific DNA-binding TCF/LEF proteins in the promoter region of targeted genes, followed by binding to chromatin remodeling complexes and transcription activator proteins. However, when Wnt/β-catenin signaling pathway is suppressed, TCF-4/LEF-1 proteins recruit structure-specific TLE1-Q tetramers to bind K20 methylated histone H4 tails to induce repression of Wnt/β-catenin targets (14). Furthermore, WNT10B activation upregulates high-mobility group AT-hook (HMGA) expression, which interacts with Enhancer of Zeste 2 (EZH2) to displace TLE1 from TCF-4 and promote K49 acetylation for transcription (23). Ramasamy et al. described that TLE1 knockdown led to increased phosphorylation and activation of NF-κB and decreased the negative inflammation regulator hairy and enhancer of split-1 (HES1), suggesting a critical role for TLE1 in NF-κB inhibition (21). Moreover, TLE1 that has been phosphorylated downstream of the RAS/extracellular signal-regulated protein kinase (ERK) pathway is excluded from the nucleus, disrupting the repressor function of TLE1 (26).

Recently, owing to its effects on various signaling pathways, the mechanisms for TLE1 in tumorigenesis have gradually been recognized. Yao et al. conducted a series of studies on TLE1 in lung cancer that proved TLE1 was an oncogene that not only sequestered mitochondrial BCL-2 inhibitor of transcription 1 (BIT1) in the nucleus, thereby blocking the anoikis function of BIT1, but also enhanced EMT through ZEB1-mediated E-cadherin repression (27, 28). However, in hepatocellular carcinoma, TLE1 can serve as a tumor suppressor. A study in Taiwan demonstrated that miRNA-657 bound to the 3′-UTR of TLE1, thereby blocking its inhibitory effect on NF-κB and promoting the invasive ability and spheroid formation capacity of liver cancer cells (29). Moreover, TLE1 has been regarded as a prognostic biomarker in several tumor types and is also related to several clinicopathological features of patients. Morrell et al. highlighted that TLE1 is associated with spindle cell morphology and is a prognostic factor for malignant melanoma (30). Brassesco et al. indicated that TLE1 mRNA was downregulated in pediatric acute lymphoblastic leukemia, where lower TLE1 expression was associated with prognostic features such as diagnostic age, absence of the common acute lymphoblastic leukemia antigen (CALLA), and high white cell count (31). In invasive breast cancer, TLE1 was shown to be significantly associated with the human epidermal growth factor receptor 2+ and triple-negative breast cancer subtypes. However, this study did not find a significant association between TLE1 expression and disease-free survival (DFS) or overall survival (OS) (32).

To the best of our knowledge, associations between TLE1 expression and the clinicopathological variables and prognosis of patients with resectable PDAC have not been explored. In our present study, high TLE1 expression was significantly associated with benign tumor behavior, and absent vascular invasion though TLE1 expression in tumor tissues was higher than in para-tumor tissues. High TLE1 expression was also significantly associated with better DSS. Moreover, in subgroup univariate analyses, higher TLE1 expression was significantly associated with better prognosis in 16 subgroups (16/24), reflecting a significant role of TLE1 in the prognosis prediction of PDAC. At the same time, TCGA database also indicated higher TLE1 expression in tumor tissues than in para-tumor tissues in PDAC (179 tumors vs. 171 para-tumor samples), although there was no significant association with better prognosis in patients with high TLE1 expression. Furthermore, *in vitro* cell biology experiments showed that TLE1 overexpression not only impaired cell migration and invasion but also inhibited cell proliferation by impeding G0/G1 transition, which consolidated the tumor-suppressing role of TLE1 in PDAC. The same contradiction for TLE1 expression also appeared in a study of gastric cancer by Lee et al. This study indicated that TLE1 expression in gastric cancer tissues was significantly higher than in non-neoplastic gastric mucosa and that higher tumor TLE1 expression was associated with a better prognosis (33). Through literature searching, we discovered that this phenomenon exists not only for TLE1 but also for several other proteins. Li and his colleagues indicated that carbonic anhydrase 12 (CA12), a transmembrane protease, showed significantly higher expression in breast cancer tissues than in para-tumor tissues. Nevertheless, higher CA12 expression was associated with significantly better prognosis of breast cancer patients for DFS and OS. The authors hypothesized that the reason may be that the hypoxic tumor environment leads to CA12 overexpression in tumor tissues (34). A study of colon adenocarcinoma using TCGA database showed that cadherin 3 (CDH3) was significantly upregulated in colon adenocarcinoma compared with para-tumor tissues, but the survival analysis showed that higher CDH3 expression was associated with a favorable survival rate (35). Xu et al. also reported that overexpression of the receptor frizzled 1 (FZD1) was detected in renal cancer tissue, renal cancer cell lines, and corresponding sunitinib-resistant cells. However, alterations in FZD1 in renal clear cell carcinoma were associated with better OS and DFS. The authors speculated that genetic mutations and epigenetic alterations may contribute to this contradictory phenomenon (36).

In this study, despite higher TLE1 expression in tumor tissues than in para-tumor tissues, both *in vitro* experiments and clinicopathological and prognostic analyses revealed a tumor-suppressing role for TLE1 in PDAC. Therefore, we consider TLE1 a tumor suppressor gene in PDAC, similar to liver and gastric cancer, which are both digestive system neoplasms (29, 33), and TLE1 is a prognostic indicator for better outcomes. The mechanisms for how TLE1 affects PDAC remain to be elucidated. Using RNA sequencing and bioinformatics, we identified 16 genes that are involved in various cell biological processes that may be regulated by TLE1 expression and then influence tumor progression. MMP23B is among the genes regulated by TLE1 in the 16 genes we identified. The expression of MMP family proteins has been reported to regulate metastasis of various cancers including pancreatic cancer (37). MMP23B expression is significantly different between tumor and para-tumor tissues in various cancers (38). Also, the study of Moogk et al. indicated the expression of MMP23 can blunt tumor immunity in melanoma, which implied the relationship between MMP23 and immunoregulation (39). Therefore, we can speculate that TLE1 may modulate EMT and immune cells infiltration through the regulation of MMP23B. However, the detailed mechanisms still need to be further studied. Previous studies showed that the mechanisms of TLE1 in tumor progression regulation may involve its negative modulation of Wnt/β-catenin and NF-κB pathways, which have been confirmed as core signaling pathways for tumorigenesis in PDAC (14, 21). However, the reason why TLE1 expression was higher in tumor tissues compared with para-tumor tissues in our study still remains unclear. One potential reason may be epigenetic modifications of TLE1 by other signaling pathways. It was reported that the promoter of TLE1 was hypomethylated in the intestinal epithelium of diabetic mice and that demethylation of the TLE1 promoter led to upregulated TLE1 mRNA and protein expression (40). As some of the PDAC patients also had diabetes, TLE1 overexpression in the pancreatic ductal epithelium may have an antitumor role. Another reason may be that TLE1 regulates CD8+ T cell generation. TLE proteins have been reported to have an important role in CD8+ T cell generation. Blocking all TLE family proteins expression largely hampers CD8+ T cell generation. Therefore, high TLE1 expression in PDAC tissues may relate to CD8+ T cell infiltration, which also has a tumor-suppressive role (41). However, this hypothesis remains to be confirmed.

In conclusion, our results confirmed that TLE1 expression inhibits malignant behaviors, such as cell metastasis and proliferation, and enhances G0/G1 retention in PDAC. Sixteen genes may be regulated by TLE1 expression. TLE1 overexpression was correlated with benign tumor behaviors and a better prognosis in PDAC patients. However, some limitations exist in our study. First, owing to lack of stably transfected PDAC cell lines, an *in vivo* study was not performed to confirm the results acquired in the *in vitro* study. Second, although we used RNA sequencing and bioinformatics to analyze the potential downstream targeted genes of TLE1, deeper and more direct evidences were absent in our study to confirm the specific genes regulated by TLE1 and the detailed mechanism of TLE1 in the progression of PDAC. Therefore, these questions and the contradiction between the difference in TLE1 expression between tumor and para-tumor tissues and the favorable prognosis of patients with higher tumor TLE1 expression warrant further study.

## Data Availability Statement

The raw data supporting the conclusions of this article will be made available by the authors, without undue reservation, to any qualified researcher.

## Ethics Statement

Written informed consent was obtained from the individual(s) for the publication of any potentially identifiable images or data included in this article.

## Author Contributions

YW, DY, JG, and Y-PZ managed the study design. YW and DY conducted the experiments and drafted the manuscript. ZL and WZ contributed to the pathological diagnosis and immunohistochemistry experiments. LZ and LY were involved in the statistical analyses. JL, BJ, JG, and Y-PZ reviewed the manuscript and made critical revisions for important intellectual content. JG and Y-PZ provided funding support. All authors read and approved the final version of the manuscript.

## Conflict of Interest

The authors declare that the research was conducted in the absence of any commercial or financial relationships that could be construed as a potential conflict of interest.

## Supplementary Material

Figure S1 **(A)** TLE1 expression compared between tumor and para-tumor tissues in the GEPIA database (based on TCGA). **(B)** DSS curve for all patients with high or low tumor TLE1 expression in TCGA (Kaplan-Meier Plotter online database: www.proteinatlas.org).
