# Supplementary figures and images for: Transducin-Like Enhancer of Split-1 Inhibits Malignant Behaviors in vitro and Predicts a Better Prognosis in Pancreatic Ductal Adenocarcinoma
Source: Front Oncol. 2020 May 5;10:576. doi: 10.3389/fonc.2020.00576 (PMC7214815; doi:10.3389/fonc.2020.00576)

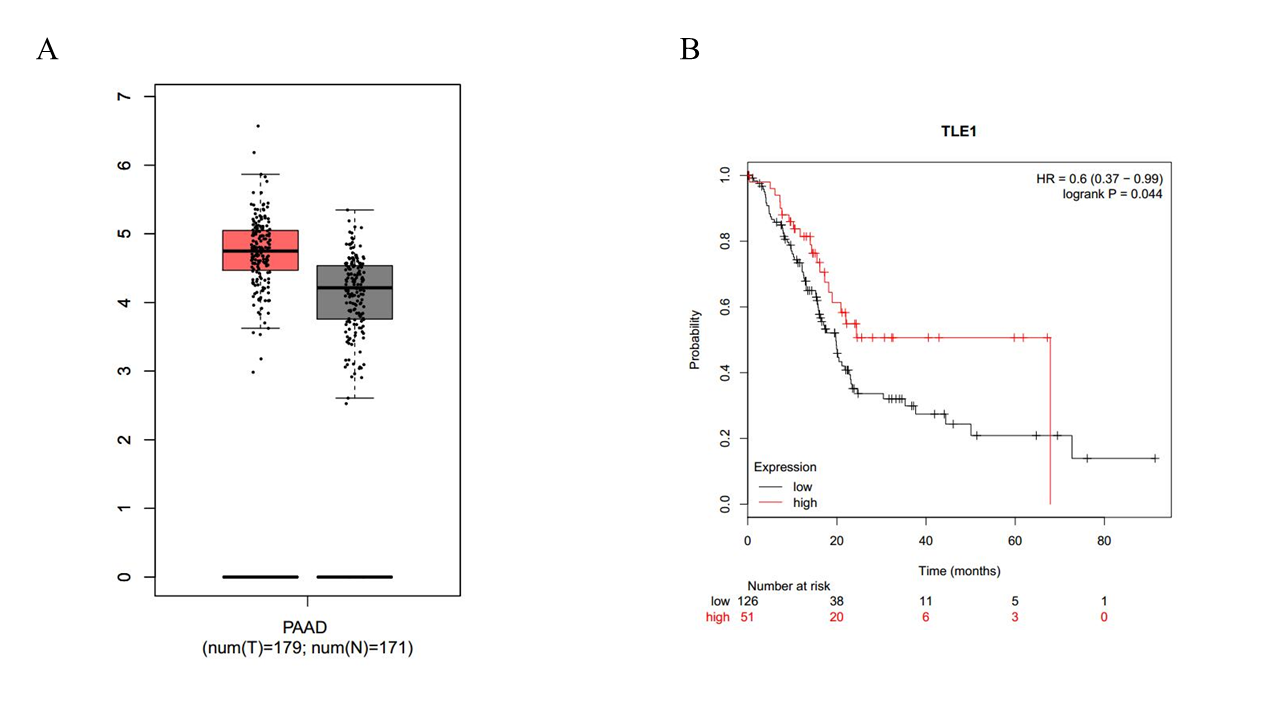

Supplement: Figure S1 — (A) TLE1 expression compared between tumor and para-tumor tissues in the GEPIA database (based on TCGA). (B) DSS curve for all patients with high or low tumor TLE1 expression in TCGA (Kaplan-Meier Plotter online database: www.proteinatlas.org). [file Image_1.tif]
